# Unusual Vulvar, Perineal, and Uterine Leiomyomas: A Case Report

**DOI:** 10.7759/cureus.43184

**Published:** 2023-08-09

**Authors:** Marta Manso, Joana M Ribeiro, Lisa Agostinho

**Affiliations:** 1 Radiology, Hospital Beatriz Ângelo, Loures, PRT; 2 Obstetrics and Gynecology, Hospital Beatriz Ângelo, Loures, PRT

**Keywords:** smooth muscle tumours, pelvic mass, uterine leiomyoma, fibroids, vulvar leiomyoma

## Abstract

Vulvar leiomyomas are extremely rare smooth muscle tumors that are easily mistaken for other lesions, as the differential diagnosis must consider a wide spectrum of benign and malignant lesions. We present the case of a 52-year-old woman with a three-year history of progressive abdominal distension and pain and an enlarging vulvar mass distorting the labia majora and causing gait disturbance. Imaging confirmed an enormous pelvic mass originating in the uterus, compatible with a leiomyoma/sarcoma, and large perineal and vulvar masses with similar characteristics. Histopathology after surgical removal revealed benign abdominal, vulvar, and perineal leiomyomas. This case highlights the rarity and diagnostic challenges of extra-uterine leiomyomas, particularly those in the vulvar region.

## Introduction

Leiomyomas are well-circumscribed, benign, smooth muscle neoplasms that most commonly present as uterine tumors in females of reproductive age. While uterine leiomyomas are fairly common, extra-uterine ones are not, and vulvar leiomyomas, in particular, are extremely rare. The unusual location and painless clinical presentation of vulvar leiomyomas present diagnostic challenges along with the fact that vulvar masses include a wide spectrum of benign and malignant tumors [1]. Ultrasound and MRI can characterize the lesions, narrow the differential diagnosis, and precisely define relations to nearby organs, but these techniques cannot safely exclude malignant transformation. Definite diagnosis requires surgical excision and histologic characterization, and long-term follow-up is recommended [2].

We report the case of a 52-year-old woman presenting with abdominal pain and anemia, exuberant vulvar and perineal masses causing gait disturbance, and a concomitant abdominal mass. Surgical excision was performed, and histologic analysis confirmed uterine, vulvar, and perineal leiomyomas.

## Case presentation

A 52-year-old multiparous female presented to the gynecological emergency department with a large vulvar mass, which had been growing for three years and was associated with gait disturbance, diffuse abdominal pain, and abdominal distension. No hormonal contraceptives were used and menstrual cycles were regular. Other symptoms, such as vaginal bleeding, fever, and recent weight loss, were not present. The patient had a medical history of high blood pressure, dyslipidemia, and previous cholecystectomy.

**Figure 1:**
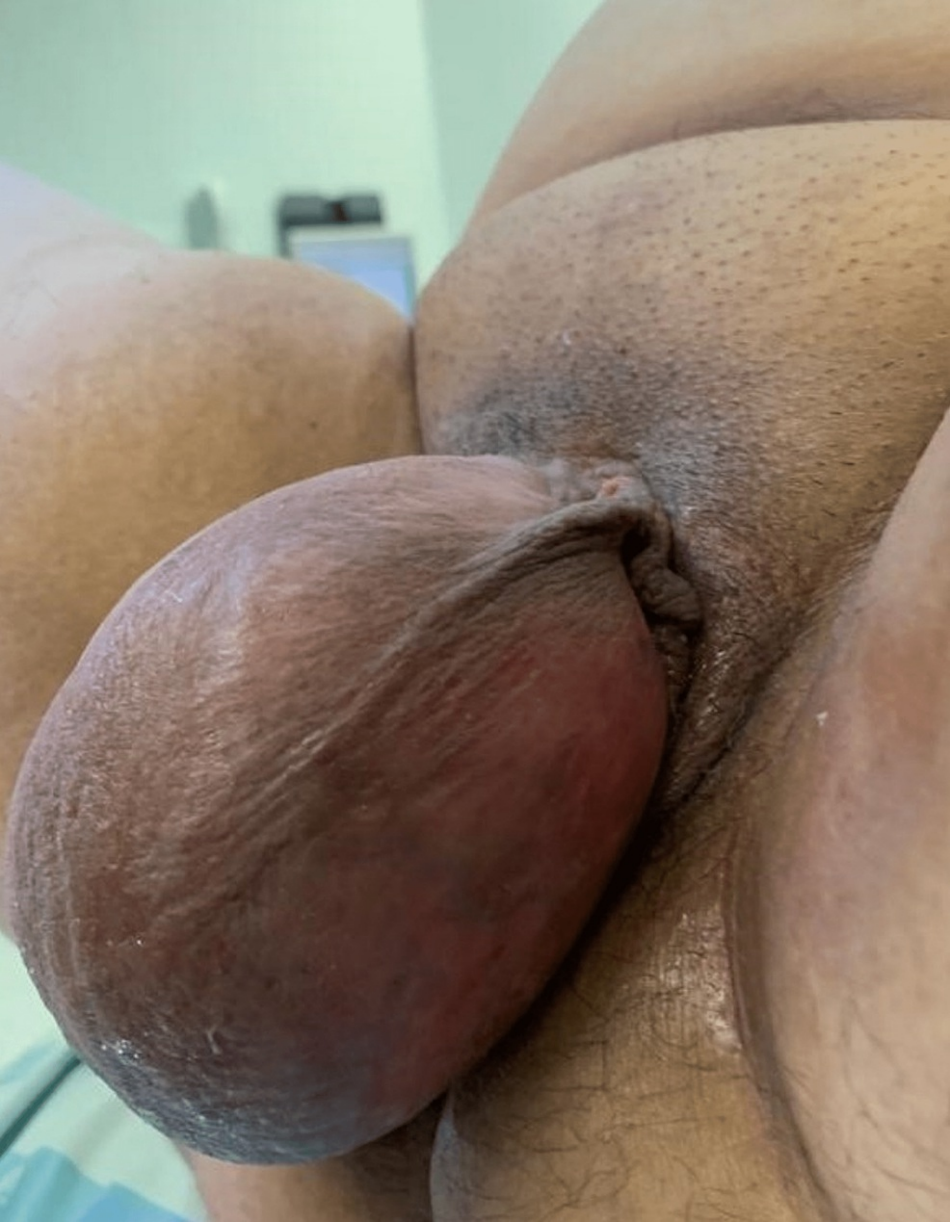
Exuberant vulvar mass causing distortion of the outer lips and gait disturbance

Gynecological examination revealed a voluminous vulvar mass distorting the labia majora. The mass was firm and somewhat mobile, without tenderness (Figure 1). Speculum evaluation was not feasible. Diffuse mild tenderness was present in the abdomen, with a palpable mass extending from the suprapubic area to the xiphoidal appendix.

**Figure 2:**
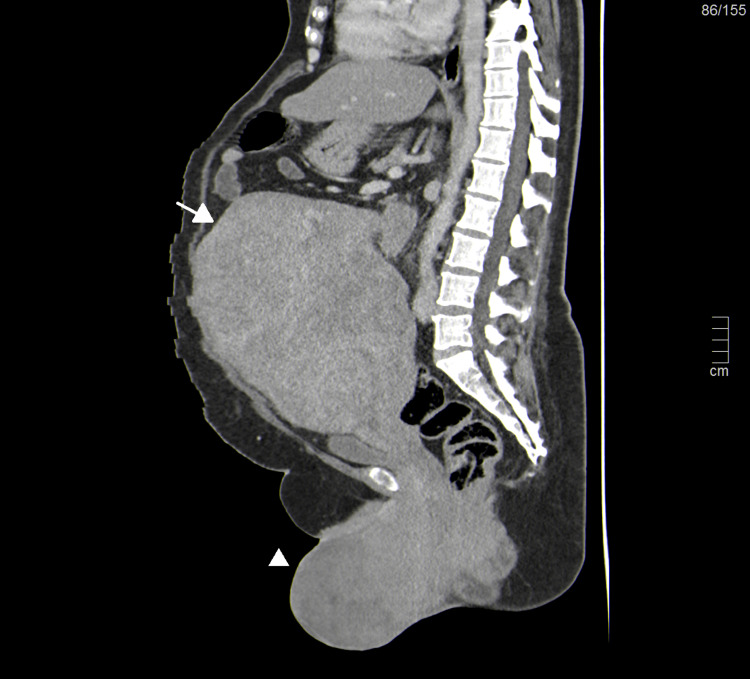
Computed tomography of the abdominal and pelvis saggital reformated plane showing enlarged abdominal (arrow) and pelvic masses (arrowhead) with regular contours and heterogeneous nodular enhancement

**Figure 3:**
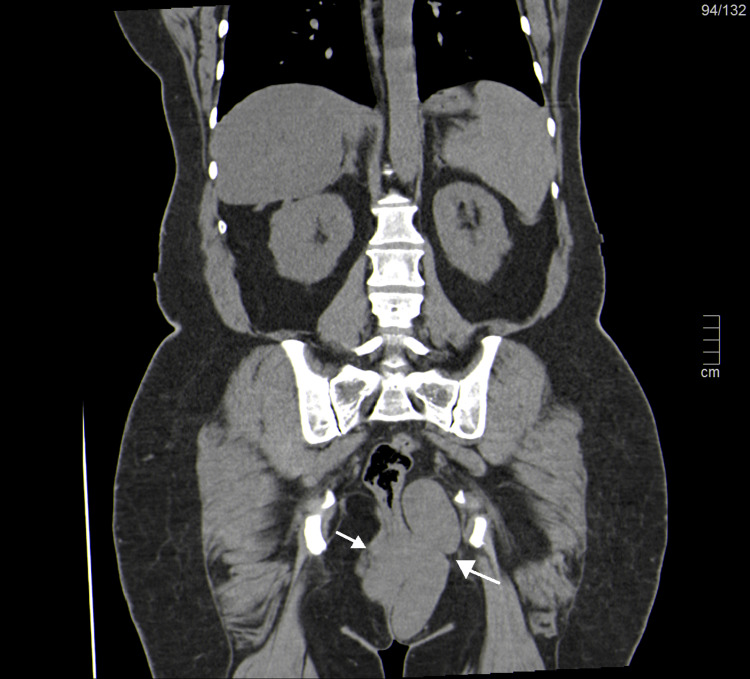
Computed tomography of the coronal reformated plane showing pelvic and vulvar masses (arrows)

Abdominal and pelvic ultrasound showed a heterogeneous mass occupying the whole pelvic and inferior abdominal cavity and a vascularized vulvar mass with similar characteristics. CT demonstrated a 24-cm pelvic mass originating in the uterus with regular contours and heterogeneous nodular enhancement, compatible with a leiomyoma/leiomyosarcoma. Numerous vulvar and perineal masses were also noted (Figures 2, 3).

**Figure 4:**
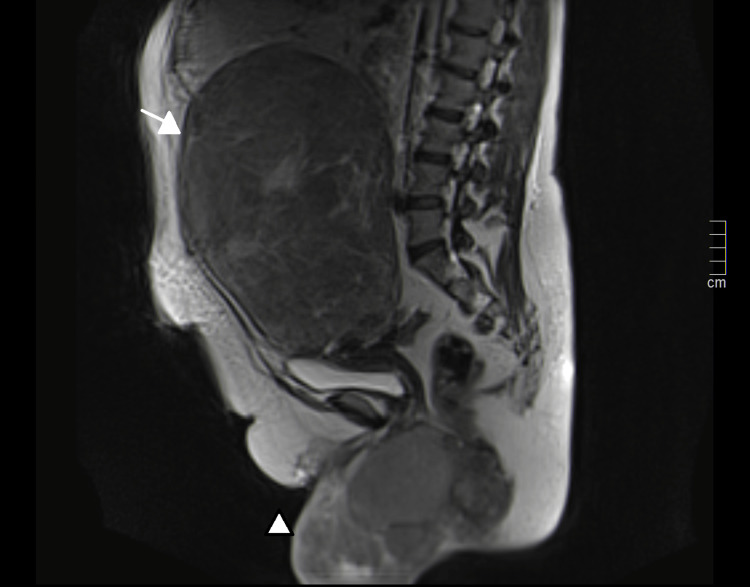
T2-weighted sagittal magnetic resonance imaging showing a hypointense uterine mass (arrow) and vulvar and perineal masses (arrowhead)

**Figure 5:**
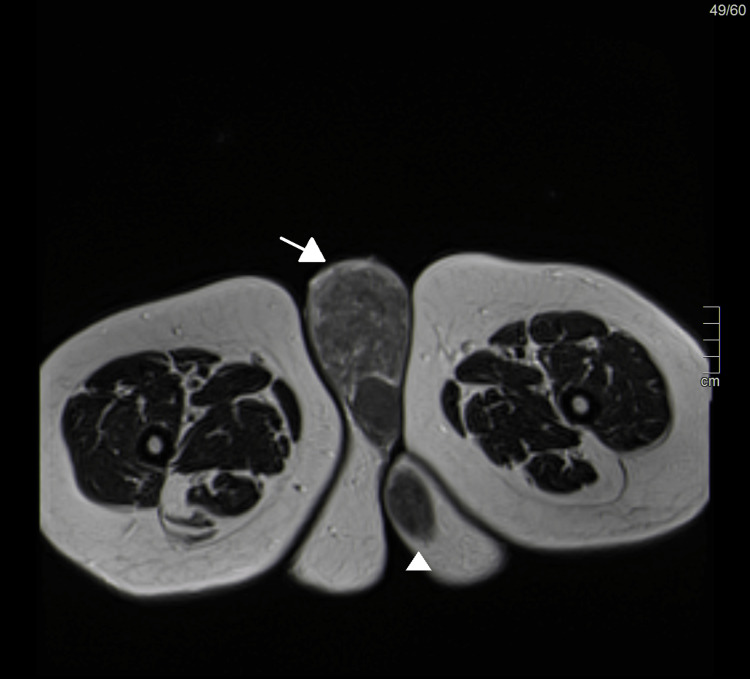
T2-weighted magnetic resonance imaging showing hypointense vulvar (arrow) and perineal masses (arrowhead)

MRI showed well-demarcated uterine, vulvar, and perineal masses, with hypointensity on T2-weighted images, absent restricted diffusion, and progressive heterogeneous enhancement on T1 post-gadolinium sequences, suggesting leiomyomas, although sarcomas could not be excluded (Figures 4, 5). The lesions did not contain calcifications or macroscopic fat.

**Figure 6:**
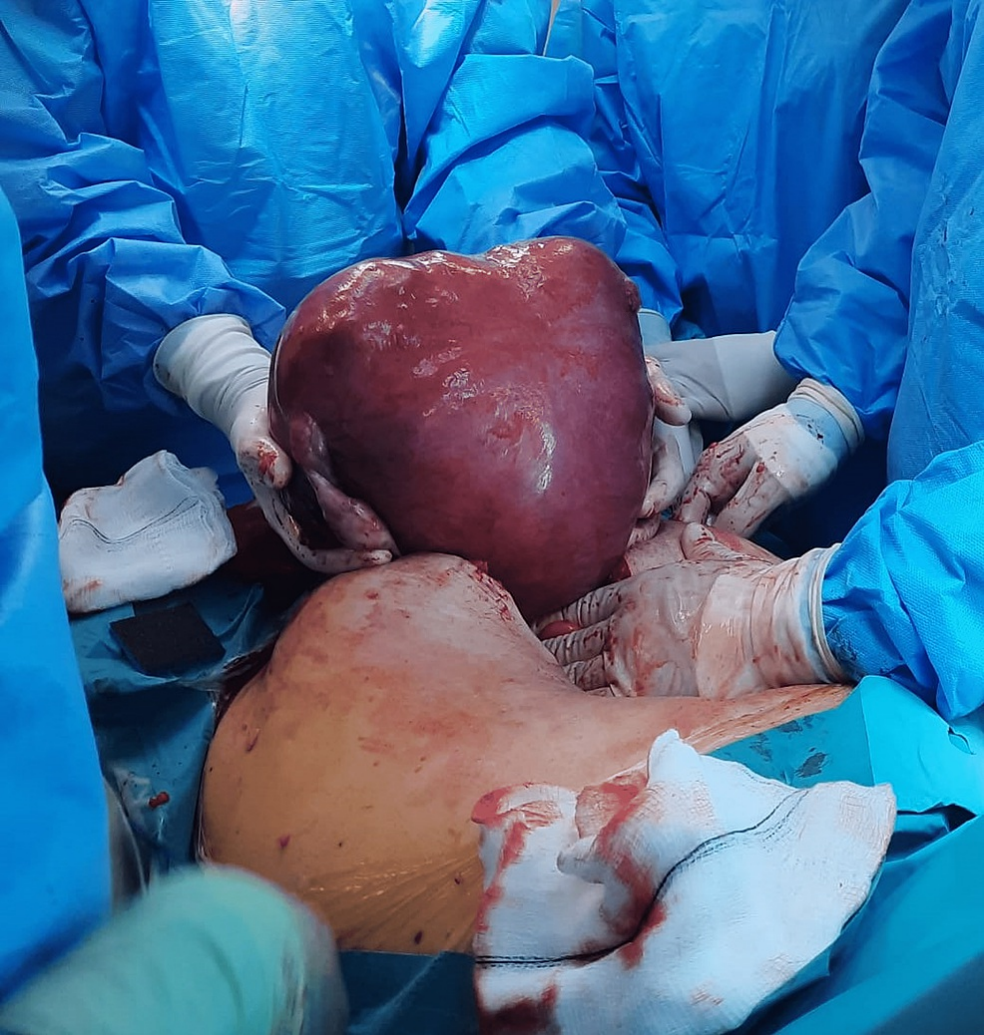
Postoperative images showing an enlarged uterus replaced by leiomyomas

**Figure 7:**
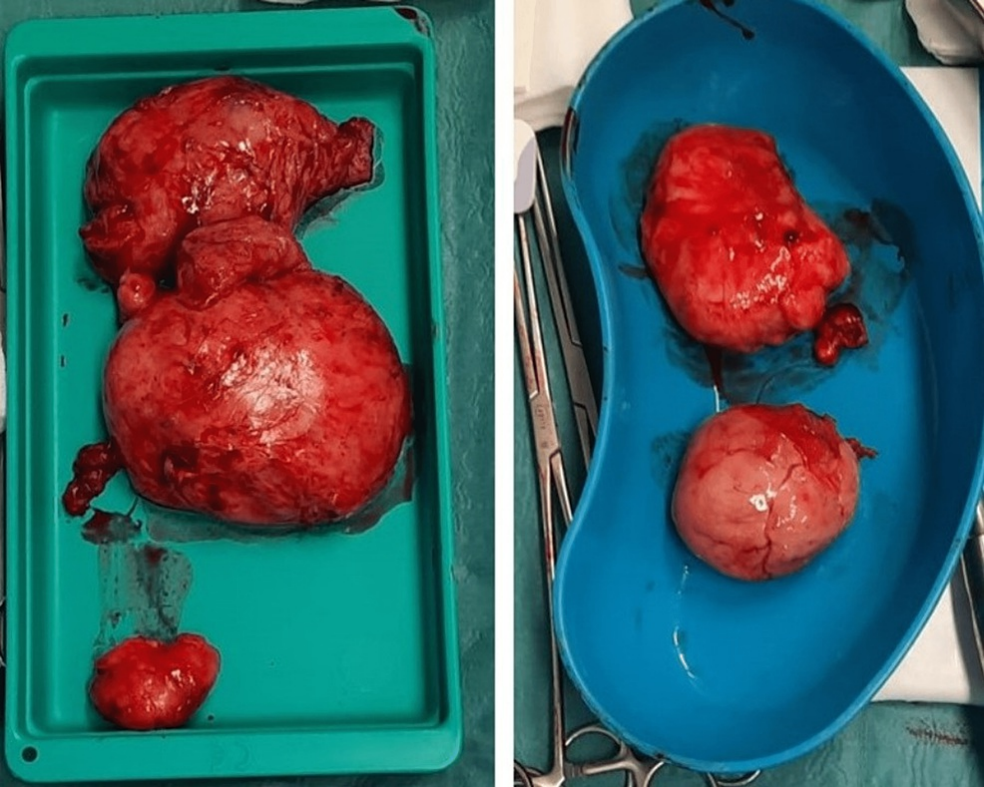
Postoperative images showing vulvar leiomyomas

The patient underwent a total abdominal hysterectomy with a bilateral adnexectomy. Intraoperatively, the uterus was markedly enlarged (30 cm) and appeared to be totally occupied by fibroids. The right vulvar masses measuring up to 10 cm and the left perineal masses measuring up to 7 cm were also removed (Figures 6, 7).

Pathology revealed a uterus replaced by leiomyomas and vulvar and perineal leiomyomas with no atypia or considerable mitosis. At two years follow-up, the patient is well and has had no recurrence of the disease.

## Discussion

Leiomyomas are the most common uterine neoplasms, known to affect 30% of women over the age of 35. They are benign soft tissue mesenchymal tumors derived from smooth muscle cells [1,2].

Leiomyomas are most often found in the uterus, with extra-uterine locations being rare. Vulvar leiomyomas account for only 0.03% of all gynecologic neoplasms and 0.07% of all vulvar tumors, with only about 300 cases described in the literature [2-4].

Vulvar leiomyomas originate from the smooth muscle within the round ligament, erectile tissue, and blood vessel walls [2,4]. Their etiology remains largely unknown, but estrogens and progesterone are thought to be involved, as receptors for these hormones are usually present in the lesions [1].

Most patients present with a long history of a painless mass that can sometimes compress the urinary bladder and rectum [3]. As the mass increases, patients can experience difficulty walking, sitting, or having intercourse. Pain can develop, and some patients report pruritus and erythema. The vulvar mass is usually well-circumscribed and firm and exhibits partial mobility.

Ultrasound is an accessible method to confirm the location and size of leiomyomas. MRI is valuable for planning surgery and for characterizing leiomyomas’ vascularization, soft tissue invasion, and relationships to nearby organs.

MRI usually shows low signal intensity like that of smooth muscle on T2-weighted images and low signal intensity in diffusion-weighted images. Leiomyosarcoma is the main concerning differential diagnosis and usually manifests as an ill-defined, heterogeneous myometrial mass that frequently presents with focal areas of high signal intensity on T1-weighted images corresponding to hemorrhage or necrosis, intermediate to high signal on T2-weighted images and low apparent diffusion coefficient (ADC) values on diffusion-weighted images. The frequent overlapping features make differential diagnosis difficult and MRI cannot safely exclude malignant transformation as 0.01%-0.5% of resected tumors diagnosed preoperatively as leiomyomas are confirmed as leiomyosarcomas [5,6].

Histopathological examination is the only accurate way to verify benignity and to rule out the presence of other kinds of mesenchymal vulval tumors, which can exhibit aggressive behavior [3,7]. Histologically, leiomyomas are circumscribed masses with spindle cells and myxoid stroma. Atypia is infrequent, and these tumors are positive for muscle markers. The majority are also positive for estrogen and progesterone receptors [1].

Surgical excision is the preferred approach to definitive diagnosis and the mainstay of treatment, with long-term follow-up advisable to detect recurrence [1]. Differential diagnosis focuses mainly on leiomyosarcoma and Bartholin´s cysts, the latter presenting as softer masses and inverted labia minora. Other possibilities are angiomyxoma, lymphangioma, dermatofibrosarcoma, and neurogenic tumors [2].

## Conclusions

Extra-uterine leiomyomas are rare, and an unusual vulvar lobe location poses challenges for diagnosis. Physical examination usually detects a firm mass, with ultrasound and MRI essential for planning surgery and facilitating differential diagnosis. Although benign, these leiomyomas can mimic aggressive mesenchymal vulvar tumors during observation and imaging. Therefore, surgical excision and histologic characterization are mandatory for confirming the diagnosis.

Early diagnosis along with appropriate management and follow-up depends on thorough examination and a familiarity with their extrauterine sites and typical imaging features. Raising awareness of these uncommon lesions can also contribute to effective diagnosis and management.
